# Bioinspired zinc-doped carbon dots protect against polystyrene microplastic-induced spermatogenic dysfunction through Nrf2 activation and cuproptosis suppression

**DOI:** 10.1016/j.mtbio.2026.103675

**Published:** 2026-09-15

**Authors:** Tushuai Li, Kehui Li, Shiqing Yang, Hongying Pan, Zhihui Cai, Xiyao Hua, Guangfu Liao, Bing Luo, Jie Zhang

**Affiliations:** a School of Biology and Food Engineering, Suzhou University of Technology, Suzhou, Jiangsu, 215500, China; b School of Life Science, Jiangsu Normal University, Xuzhou, Jiangsu, 221116, China; c College of Materials Engineering, Fujian Agriculture and Forestry University, Fuzhou, 350002, China

**Keywords:** Carbon dots, Trifolirhizin, Microplastics, Oxidative stress, Copper homeostasis, Male reproductive injury

## Abstract

Polystyrene microplastics (PS-MPs) are increasingly recognized for their detrimental effects on reproductive health, particularly in the male reproductive system. While mitochondrial oxidative stress is recognized as a driver of MPs-induced reproductive injury, it remains unclear whether this process involves cuproptosis, a newly identified copper-dependent form of regulated cell death, and elucidating this potential link is critical for alleviating such damage. Here, we report that PS-MPs exposure induces testicular injury and spermatogenic dysfunction in mice, associated with redox imbalance, copper transport defects, and upregulation of the cuproptosis regulators Ferredoxin 1 (FDX1) and Lipoic Acid Synthetase (LIAS). In turn, zinc-doped trifolirhizin-derived carbon dots (ZnTFZCDs) were rationally designed via a one-step hydrothermal strategy, embedding the antioxidant properties of a natural flavonoid and the reproductive-protective role of zinc into a biocompatible carbon nanoplatform. ZnTFZCDs are well-dispersed carbon dots rich in oxygen/nitrogen-containing groups with Zn coordination, and demonstrate favorable biosafety without overt cytotoxicity, hemolysis, or organ damage. *In vitro*, ZnTFZCDs potently scavenge intracellular reactive oxygen species (ROS), maintain antioxidant enzyme activities, preserve mitochondrial membrane potential, suppress lipid peroxidation, and rescue Glutathione peroxidase 4 (GPX4) expression in MPs-exposed RAW264.7 and GC-2 cells. Notably, ZnTFZCDs modulate copper homeostasis by upregulating the copper exporter ATP7B, downregulating the importer SLC31A1, and reducing FDX1/LIAS expression, thereby inhibiting cuproptosis-related signaling. *In vivo*, ZnTFZCDs administration ameliorates testicular histopathology, improves sperm count and morphology, reactivates the Nrf2/HO-1 antioxidant axis, and suppresses cuproptosis-associated alterations in testicular tissues, confirming the translational potential of the *in vitro* findings. Collectively, ZnTFZCDs represent a bio-inspired nanoplatform that attenuates PS-MPs-induced male reproductive injury through coordinated modulation of redox and copper homeostasis, offering a new therapeutic strategy for environmental pollutant-related reproductive disorders by targeting the previously overlooked cuproptosis pathway.

## Highlights

• A zinc-doped trifolirhizin-derived carbon dots was developed.
• ZnTFZCDs demonstrate favorable biosafety without overt cytotoxicity, hemolysis, or organ damage.
• ZnTFZCDs administration ameliorates testicular histopathology, improves sperm count and morphology.
• ZnTFZCDs reactivates the Nrf2/HO-1 antioxidant axis, and suppresses cuproptosis-associated alterations in testicular tissues.

## Graphical abstract

This work reports a novel zinc-doped trifolirhizin-derived carbon dots (ZnTFZCDs) for attenuating PS-MPs-induced male reproductive injury through coordinated restoration of redox and copper homeostasis.

**Figure:**
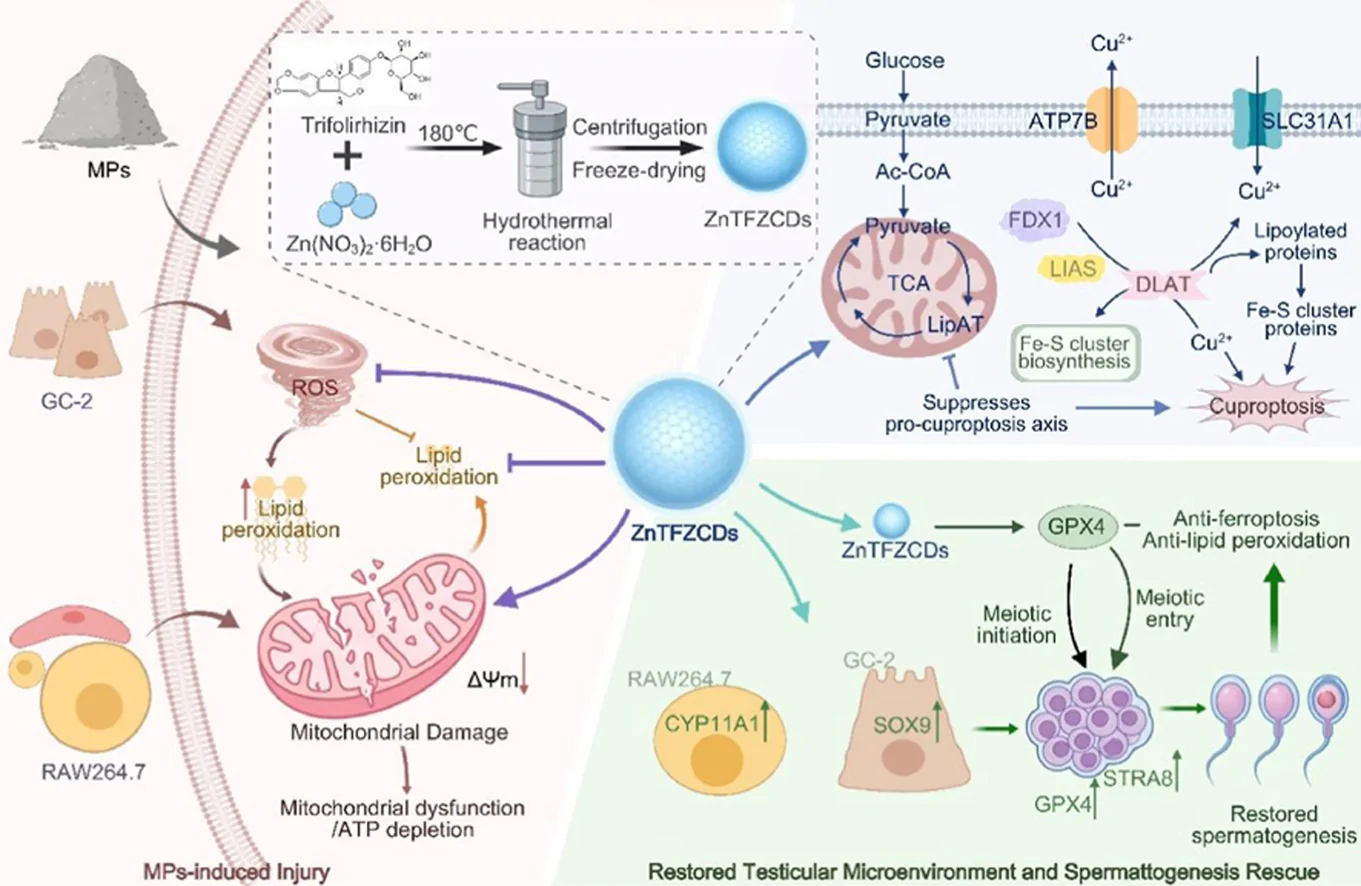

## 1 Introduction

**Scheme 1:**
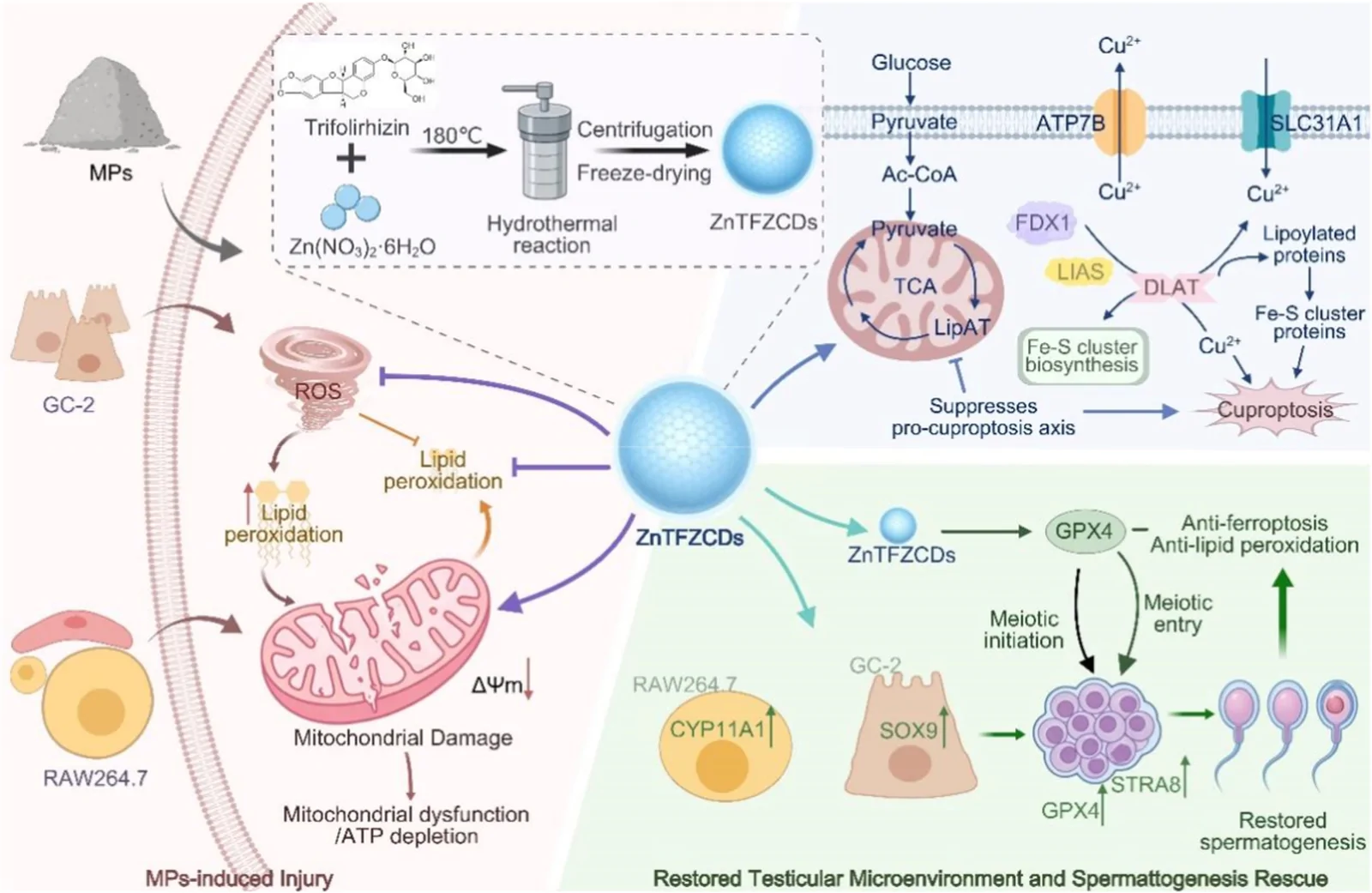
Schematic illustration of the protective mechanism of ZnTFZCDs against MPs-triggered male reproductive injury via regulating copper homeostasis as well as mitochondrial oxidative damage.

The escalating accumulation of environmental pollutants has become a major threat to both ecological and public health, especially as its association with declining fertility has drawn growing concern [1,2]. Due to their abundance, persistence, and bioavailability, polystyrene microplastics (PS-MPs) are ubiquitous environmental pollutants that inevitably expose humans through ingestion of contaminated food and water [3]. Accumulating evidence has demonstrated that MPs exposure induces male reproductive toxicity, manifested as testicular histopathological damage, reduced sperm count and motility, increased sperm malformation rates, and disrupted testosterone synthesis [4]. Mechanistically, oxidative stress has been identified as a critical driver of this reproductive injury [2]. MPs exposure promotes excessive production of reactive oxygen species (ROS), depletes antioxidant enzyme superoxide dismutase (SOD), Catalase (CAT), glutathione peroxidase (GPx) activities, elevates lipid peroxidation markers such as malondialdehyde (MDA), and activates stress-responsive signaling pathways including p38 MAPK and NF-κB, ultimately leading to spermatogenic cell apoptosis and impairment of the blood-testis barrier (BTB) [[5], [6], [7]] (see Scheme 1).

Despite the well-established role of oxidative stress in MPs-induced reproductive toxicity, the full spectrum of cell death pathways involved remains incompletely understood. Cuproptosis, a recently identified form of regulated cell death, is mechanistically distinct from apoptosis, ferroptosis, and necroptosis [8]. It is triggered by intracellular copper overload and depends on mitochondrial respiration, particularly the direct interaction of copper with lipoylated proteins in the tricarboxylic acid cycle, which results in lipoylated protein aggregation, loss of iron-sulfur cluster proteins, proteotoxic stress, and eventual cell death [8]. Given the established link between copper homeostasis and mitochondrial oxidative stress in cardiovascular [9], central nervous [10], musculoskeletal [11], retinal [12], and mammary systems [13], and that MPs-induced reproductive toxicity involves both redox imbalance and mitochondrial dysfunction [14], it is plausible that oxidative stress may consequently trigger cuproptosis and contribute to testicular injury. Elucidating this possibility may therefore extend the current understanding of MPs-induced male reproductive toxicity beyond conventional oxidative damage models.

Current therapeutic strategies against MPs-induced reproductive injury remain largely limited to conventional antioxidant supplementation [15]. However, antioxidant monotherapy often yields unsatisfactory efficacy when oxidative stress is accompanied by metal-mediated mitochondrial damage and aberrant regulated cell death pathways [16,17]. In this context, natural bioactive molecules, which possess multi-targeting antioxidant and anti-inflammatory properties, have emerged as promising alternatives for redox-oriented intervention [18]. Trifolirhizin (TFZ), a naturally occurring flavonoid glycoside, has been reported to possess potent antioxidant and anti-inflammatory properties, as evidenced by its ability to reduce ROS production and suppress pro-inflammatory cytokines [18]. Nevertheless, the direct application of free natural molecules is constrained by limited stability, poor bioavailability, and insufficient subcellular targeting capacity [19]. Carbon dots (CDs), as ultrasmall carbon nanomaterials, have emerged as versatile platforms for biomedical applications due to their excellent biocompatibility, tunable surface chemistry, and intrinsic antioxidant activities [20,21]. CDs derived from natural medicinal plant sources are considered particularly attractive, as they retain bioactive functionalities of their precursors while offering enhanced stability and cellular uptake [22,23]. Zinc (Zn) is an essential trace element for male reproductive health, with established roles in testicular development, spermatogenesis, sperm membrane stabilization, and hormone regulation [[24], [25], [26]]. As Zn exists as Zn^2+^ and is redox-inert, it does not directly generate oxidative stress, unlike redox-active metals such as iron and copper [27]. Zn supplementation has been shown to protect against environmental toxicant-induced reproductive damage by alleviating oxidative stress and inhibiting ferroptosis and apoptosis [28,29]. Furthermore, given the competitive antagonism between zinc and copper in intestinal absorption and metal transporter binding [30], Zn may also modulate cuproptosis through copper homeostasis, a hypothesis that warrants further investigation in male reproductive toxicology.

Based on the above considerations, we rationally designed and synthesized zinc-doped trifolirhizin-derived carbon dots (ZnTFZCDs) via a one-pot hydrothermal strategy. In this study, we aim to systematically characterize the physicochemical properties of ZnTFZCDs and evaluate their protective effects against MPs-induced reproductive injury, using GC-2 spermatocytes and RAW264.7 macrophages for *in vitro* assays and a mouse model for *in vivo* validation. We further investigate whether ZnTFZCDs restore redox homeostasis by scavenging ROS, preserving mitochondrial function, and upregulating antioxidant defenses, and determine whether they re-establish copper homeostasis by modulating copper transporters ATP7B/SLC31A1 and cuproptosis regulators FDX1/LIAS. The comprehensive biosafety profile of ZnTFZCDs is finally assessed to support their translational potential. By integrating mechanistic investigation with rational nanomaterial design, this study is expected to advance the understanding of MPs-induced male reproductive toxicity and may offer a new therapeutic strategy for precision intervention in environment-related reproductive disorders.

## 2 Results and discussion

### 2.1 MPs trigger testicular injury, copper homeostasis and suppressed Nrf2 antioxidant signaling in a dose-dependent manner

**Fig. 1:**
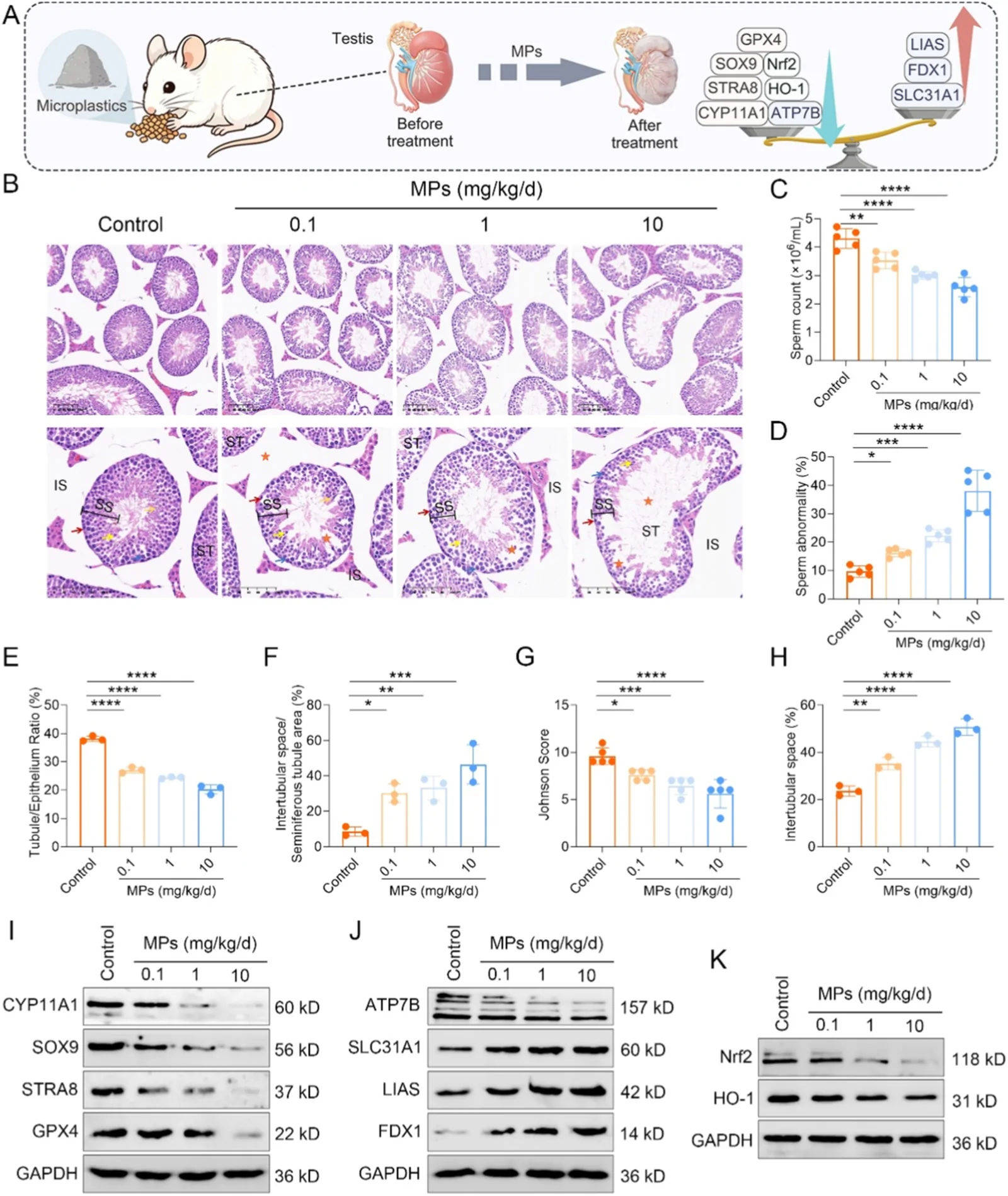
**MPs exposure induces testicular histopathological damage, spermatogenic dysfunction, oxidative stress, and copper homeostasis disturbance in mice.** (A) Schematic illustration of the animal experimental timeline. (B) Representative H&E staining of testicular sections from mice exposed to MPs at 0, 0.1, 1, and 10 mg/kg/day for 8 consecutive weeks. Asterisks indicate representative histopathological alterations in seminiferous tubules. IS, interstitial space; SS, seminiferous spermatogenic cell layer; ST, seminiferous tubule. (C-D) Quantitative statistical analysis of total sperm count (C) and sperm abnormality rate (D). (E-H) Morphometric quantification of testicular pathological indexes: seminiferous tubule-to-epithelium area ratio (E), relative intertubular space area (F), Johnson's spermatogenic function score (G), and intertubular space percentage (H). (I-K) Western blot analysis of three groups of target proteins: reproductive injury-related proteins CYP11A1, SOX9, STRA8, and GPX4 (I); cuproptosis-related proteins ATP7B, SLC31A1, LIAS, and FDX1 (J); oxidative stress-related proteins Nrf2 and HO-1 (K). Data are presented as mean ± SD, n = 5 for (C, D, G): sperm-related indices and Johnson scoring with high inter-mouse variability; n = 3 for (E, F, H): histomorphometric quantification from randomly-selected fields of testicular sections. Statistical significance is indicated as *P < 0.05, **P < 0.01, ***P < 0.001, and ****P < 0.0001.

To systematically characterize male reproductive toxicity induced by MPs, an *in vivo* mouse model was established via drinking administration of 0.1, 1, and 10 mg/kg/d MPs treatment for 8 consecutive weeks, aiming to simulate chronic daily human exposure scenarios (Fig. 1A). Histopathological examination revealed intact seminiferous tubule architecture, ordered stratification of spermatogenic cells and well-organized intertubular space in the control group (Fig. 1B). With increasing MPs dosage, progressive pathological alterations emerged, including loosening and disorganization of the seminiferous epithelium, germ cell exfoliation, vacuolization, and enlargement of seminiferous and interstitial spaces. Quantitative morphological statistics further confirmed these histological changes, showing dose-dependent decreases in tubule-to-epithelium ratio and Johnson's score, alongside marked interstitial area elevation at 1 and 10 mg/kg/d doses (Fig. 1E–H). Consistently, MPs exposure significantly impaired sperm quality, as evidenced by a significant decline in sperm count accompanied by a progressive increase in sperm abnormality rate (Fig. 1C and D). The dose-dependent impairments of testicular integrity and spermatogenesis by MPs likely involve their systemic translocation to the testis and BTB disruption culminating in seminiferous epithelial damage, germ cell loss, and spermatogenic failure, a link supported by population evidence that MPs in human semen are negatively associated with sperm concentration and motility [31,32].

To reveal the molecular basis underlying MPs-induced spermatogenic impairment, we performed western blot detection for key proteins governing spermatogenesis progression. MPs exposure dose-dependently downregulated Sertoli/BTB homeostatic SOX9, meiotic initiatory STRA8, and testosterone-synthetic CYP11A1 (Fig. 1I–S1). Notably, copper homeostasis disruption by MPs treatment was evidenced by upregulated influx transporter SLC31A1 and elevated cuproptosis executors LIAS and FDX1 alongside downregulated efflux chaperone ATP7B (Fig. 1J–S2), with the coordinated transporter changes driving copper overload and labile pool expansion to prime cuproptosis [33]. Consistent with this cascade, downregulated antioxidative GPX4 was observed in MPs-treated testis (Fig. 1I–S1). In addition, MPs exposure significantly downregulated Nrf2 and its downstream antioxidant effector HO-1 (Fig. 1K–S3), which constitute the master endogenous cellular defense pathway against oxidative stress and diverse regulated cell death pathways [34]. Strikingly, all toxic endpoints exhibited obvious dose-response relationships. Even at the environmentally relevant low dose of 0.1 mg/kg/d, molecular perturbations such as copper dyshomeostasis and oxidative stress were detectable without overt histological damage, indicating subclinical reproductive injury upon chronic low-level MPs exposure. Given that current global dietary MP intake estimates are comparable to this low-dose level [35], these findings highlight the hidden health hazards of chronic low-concentration MPs contamination in daily life.

### 2.2 Preparation and structural characterization of ZnTFZCDs

**Fig. 2:**
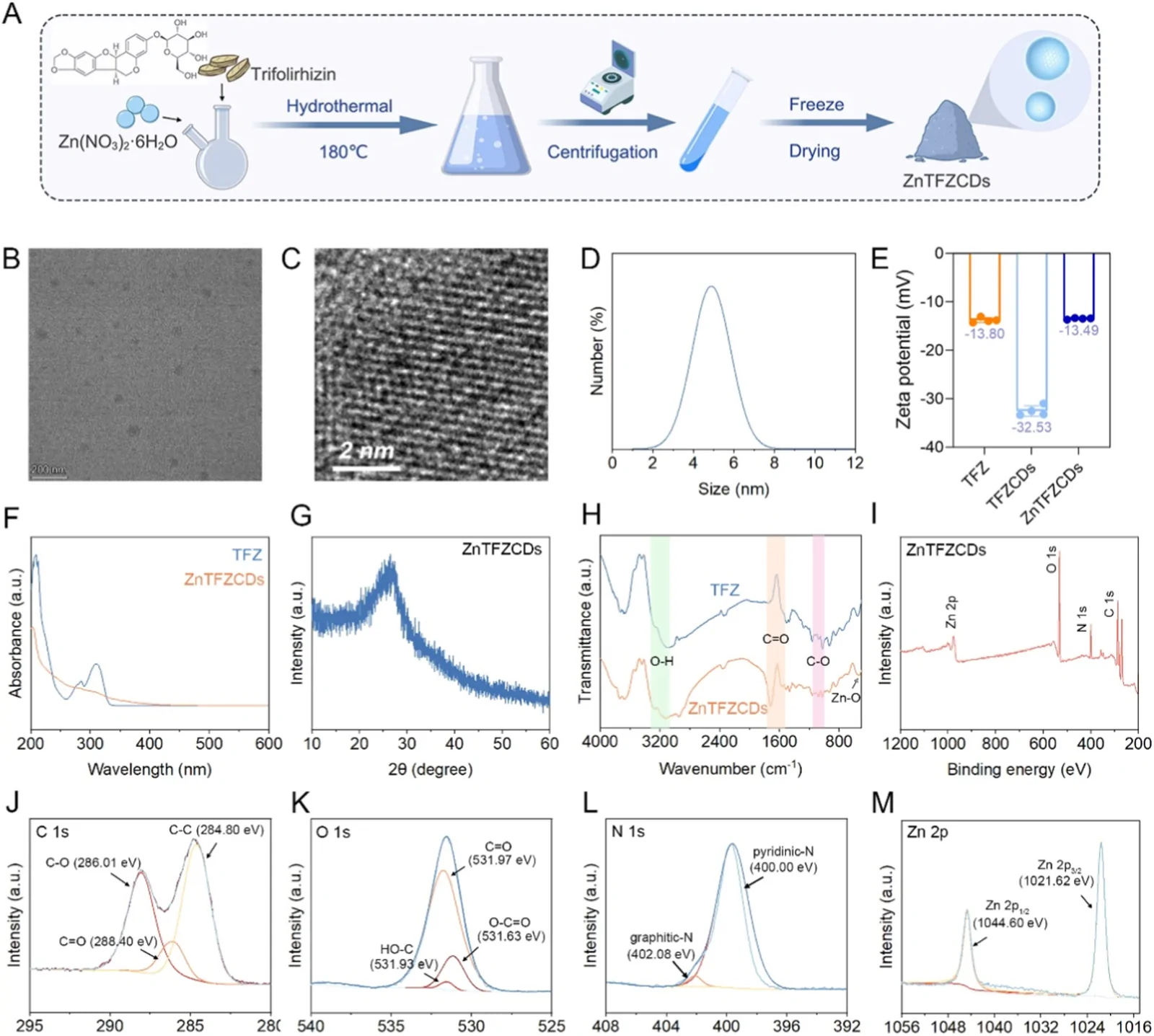
**Fabrication and physicochemical characterization of ZnTFZCDs.** (A) Schematic illustration for the hydrothermal synthesis procedure of ZnTFZCDs. (B) TEM image of ZnTFZCDs. (C) HRTEM image of ZnTFZCDs. (D) DLS hydrodynamic size distribution profile of ZnTFZCDs. (E) Zeta potentials of TFZ, TFZCDs, and ZnTFZCDs. Data are presented as mean ± SD, n = 4. (F) UV-vis absorption spectra of TFZ and ZnTFZCDs. (G) XRD pattern of ZnTFZCDs. (H) FTIR spectra of TFZ and ZnTFZCDs. (I) XPS survey spectrum of ZnTFZCDs. (J–M) High-resolution XPS spectra of C 1s (J), O 1s (K), N 1s (L), and Zn 2p (M).

Trifolirhizin (TFZ) was co-processed with zinc nitrate hexahydrate via a one-pot hydrothermal strategy to fabricate zinc-doped carbon dots (ZnTFZCDs, Fig. 2A), with the expectation that this nanoformulation would harness both the intrinsic zinc ion supplementation and the enhanced bioactivity of TFZ for synergistic mitigation against MPs-induced testicular lesions. Transmission electron microscopy (TEM) images revealed that ZnTFZCDs were well-dispersed ultrasmall spheres without obvious aggregation (Fig. 2B), with High-resolution TEM (HRTEM) further showing clear lattice fringes indicative of locally ordered carbon domains formed during hydrothermal carbonization (Fig. 2C). DLS analysis revealed a narrow hydrodynamic size distribution centered at approximately 4-6 nm (Fig. 2D), consistent with TEM observations, a uniform ultrasmall dimension favorable for aqueous dispersibility, cellular interaction, and efficient bioactivity [36]. Zeta potential detection revealed that TFZ, TFZCDs, and ZnTFZCDs all exhibited negatively charged surfaces, with TFZCDs displaying the most negative value of −32.53 mV (Fig. 2E). After zinc ion doping, the absolute zeta potential value declined to −13.49 mV for ZnTFZCDs, which originated from electrostatic interaction between exogenous Zn^2+^ and negatively charged oxygen-containing functional groups anchored on carbon dot surface [37], preliminarily verifying successful zinc element incorporation into the carbon skeleton. UV-vis absorption spectroscopy showed partial attenuation and redshift of characteristic TFZ peaks after hydrothermal carbonization and zinc doping (Fig. 2F), implying the occurrence of dehydration condensation, aromatic skeleton rearrangement, and carbonization reactions during the synthetic process. In addition, the XRD pattern of ZnTFZCDs displayed a broad diffraction peak centered in the 20-30° region (Fig. 2G), characteristic of amorphous or turbostratic carbon and indicating a predominantly disordered framework typical of biomass- or small-molecule-derived carbon dots [38].

Fourier-transform infrared spectroscopy (FTIR) was adopted to track surface functional group evolution before and after carbonization (Fig. 2H). ZnTFZCDs retained a broad absorption band spanning 3200-3600 cm^−1^, which was assigned to O-H stretching vibration, along with characteristic peaks at approximately 1700 cm^−1^ and 1600 cm^−1^ corresponded to stretching vibration of C=O and C=C, respectively. Meanwhile, a newly emerged Zn-O absorption signal at low wavenumbers, coupled with characteristic Zn 2p_3_/_2_ and Zn 2p_1_/_2_ peaks at 1021.62 and 1044.60 eV (Fig. 2M), providing solid evidence for successful zinc doping on carbon dot surface within ZnTFZCDs. X-ray photoelectron spectroscopy (XPS) full survey spectrum was acquired to analyze the elemental composition of ZnTFZCDs, which displayed dominant C, O, N elemental signals together with explicit Zn characteristic peak (Fig. 2I). High-resolution XPS deconvolution revealed that the C 1s spectrum of ZnTFZCDs was fitted into C-C, C-O, and C=O peaks (Fig. 2J), while the O 1s spectrum yielded HO-C, C=O, and O-C=O configurations (Fig. 2K), collectively confirming abundant oxygen-rich polar groups on the carbon dot surface, in good agreement with FTIR findings. The N 1s spectrum revealed pyridinic-N and graphitic-N species (Fig. 2L). Together, these results indicate that the successful fabrication of ZnTFZCDs as ultrasmall, well-dispersed, amorphous carbon dots with abundant oxygen-/nitrogen-containing functional groups and stable Zn incorporation provides a favorable structural basis for their subsequent antioxidant, cytoprotective, and cuproptosis-regulating activities observed in biological studies.

### 2.3 ZnTFZCDs rectify cellular redox homeostasis and oxidative damage cascade

**Fig. 3:**
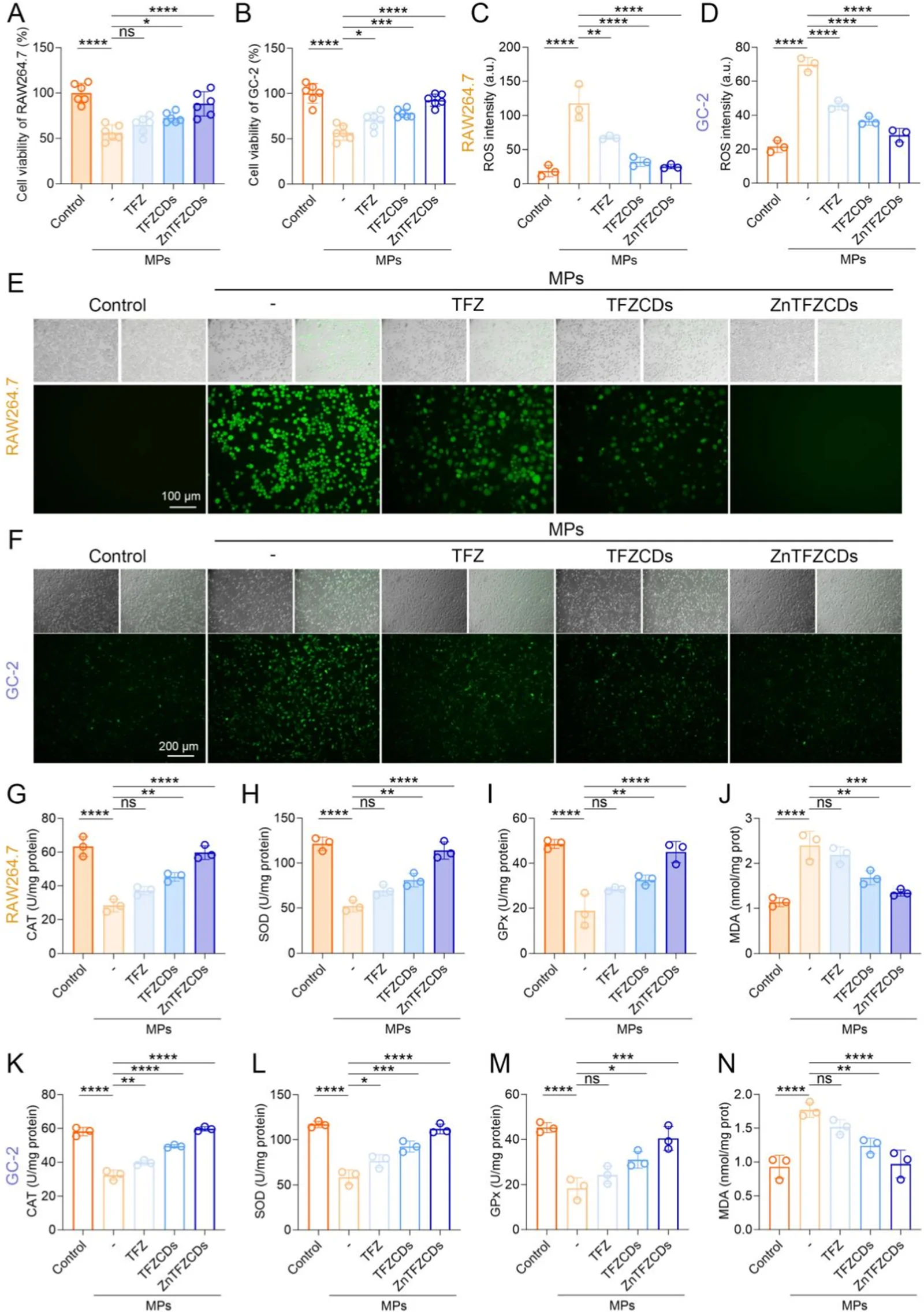
**ZnTFZCDs alleviate MPs-induced cytotoxicity and excessive oxidative stress in RAW264.7 and GC-2 cells.** (A, B) Cell viability of RAW264.7 (A) and GC-2 cells (B). (C, D) Quantitative analysis of intracellular ROS levels in RAW264.7 (C) and GC-2 cells (D). (E, F) Representative phase-contrast images (upper panels, which show general cellular morphology for intuitive evaluation of cell injury and protective effects) and ROS immunofluorescence images (lower panels) of RAW264.7 (E) and GC-2 (F) cells. Scale bar = 100 μm for RAW264.7, 200 μm for GC-2. (G-J) Levels of CAT (G), SOD (H), GPx (I), and MDA (J) in RAW264.7 cells. (K-N) Levels of CAT (K), SOD (L), GPx (M), and MDA (N) in GC-2 cells. Data are presented as mean ± SD, biological replicates: n = 6 for A, B; n = 3 for C, D, G-N. Statistical significance is indicated as ns, not significant; *P < 0.05; **P < 0.01; ***P < 0.001; ****P < 0.0001.

RAW264.7 macrophages and GC-2 spermatocytes, two widely used *in vitro* models for assessing MPs-induced oxidative stress/immune responses and reproductive toxicity, respectively [14,39], were selected to preliminarily validate the cytoprotective efficacy of ZnTFZCDs against MPs toxicity at the cellular level. Cell viability assays were first performed to assess the protective effect of test samples against MPs-elicited cell proliferation inhibition (Fig. 3A and B). Compared with the blank Control group, single MPs incubation significantly reduced the viability of both RAW264.7 and GC-2 cells. Co-treatment with free TFZ alone only yielded marginal restoration of cell viability, TFZCDs displayed moderate cytoprotective potency, whereas free Zn^2+^ exerted only slight improvement (Fig. S4). Notably, ZnTFZCDs intervention produced the most pronounced rescuing effect and reversed MPs-induced cell-viability decline in both cell lines in a concentration-dependent manner, with maximal rescue efficacy achieved at 100 μg/mL (Fig. S5). Moreover, we explored whether such protection is specific to PS-MPs, GC-2 cells were challenged with polyethylene microplastics (PE-MPs). ZnTFZCDs significantly improved cell viability under PE-MPs exposure with weaker rescue efficacy than for PS-MPs (Fig. S6), indicating broad but variable cytoprotection against different microplastics, though further mechanistic studies are needed for PE-MPs.

Excessive reactive oxygen species (ROS) burst is a core upstream event mediating MPs-triggered cellular oxidative damage and subsequent redox imbalance [2]. Quantitative and visual ROS data (Fig. 3C–F) confirmed that MPs induced severe oxidative burst in RAW264.7 and GC-2 cells, with ZnTFZCDs exhibiting the most potent ROS-scavenging effect, followed by TFZCDs and then free TFZ. Consistently, quantitative detection of catalase (CAT), superoxide dismutase (SOD), glutathione peroxidase (GPx) activities and malondialdehyde (MDA) content in both cell lines revealed that MPs exposure significantly depleted these antioxidant enzymes and elevated MDA levels, indicating severe antioxidant system collapse and aggravated lipid peroxidation (Fig. 3G–N). Among the interventions, ZnTFZCDs markedly rescued enzyme activities and reduced MDA accumulation, outperforming both TFZ and TFZCDs.

### 2.4 ZnTFZCDs break the mitochondrial-driven lipid peroxidation cascade

**Fig. 4:**
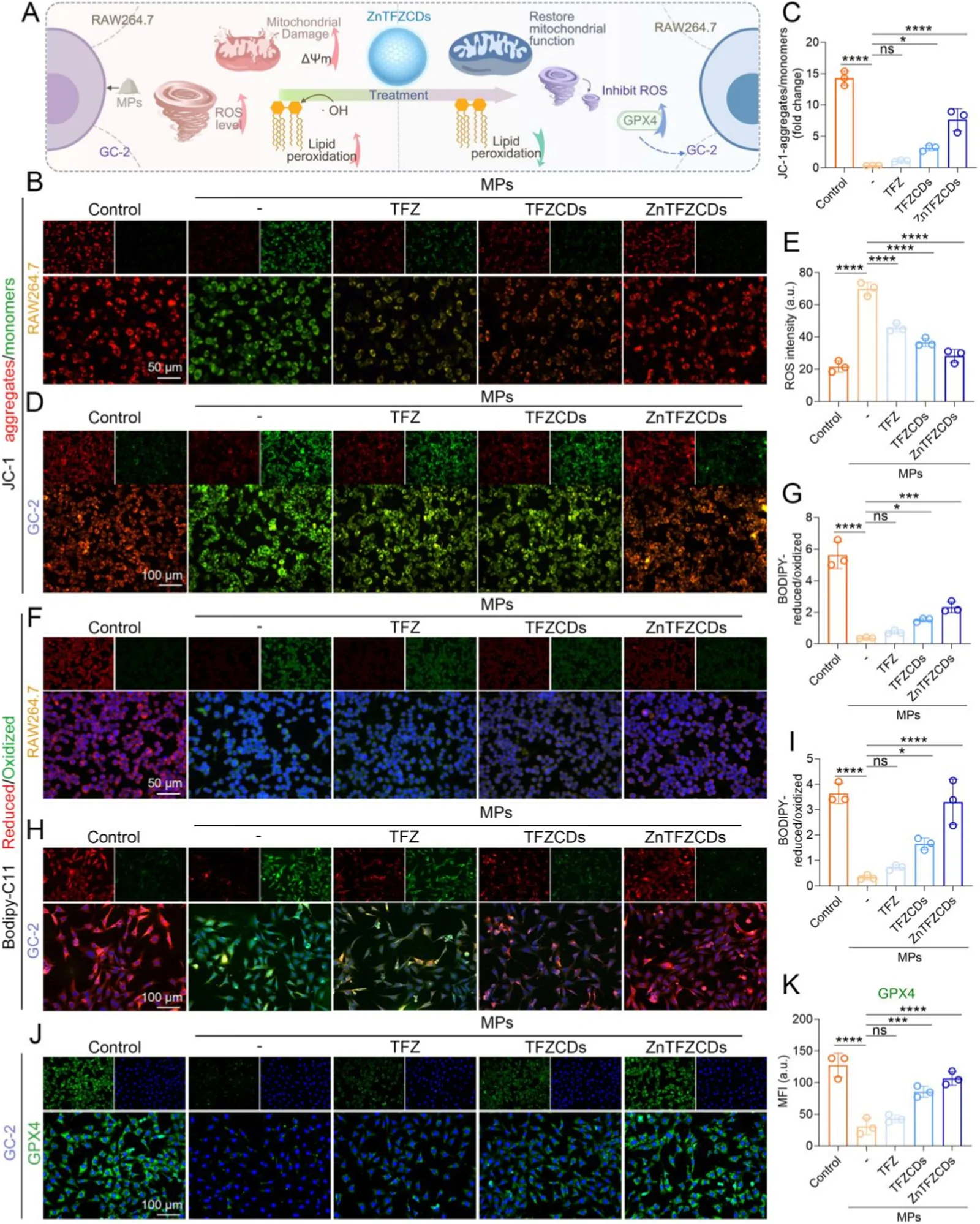
**ZnTFZCDs rescue mitochondrial depolarization, restrain lipid peroxidation and maintain GPX4 abundance in MPs-exposed RAW264.7 and GC-2 cells.** (A) Schematic illustration of the proposed mechanism by which ZnTFZCDs alleviate MPs-induced oxidative injury. (B, D) JC-1 fluorescence images reflecting ΔΨm in RAW264.7 (B) and GC-2 (D) cells under different treatments. Scale bar = 50 μm for RAW264.7, 100 μm for GC-2. (C, E) Quantitative statistical analysis of JC-1 aggregates/monomers fluorescence ratio in RAW264.7 (C) and GC-2 (E) cells. (F, H) BODIPY-C11 immunofluorescence staining for intracellular lipid peroxidation in RAW264.7 (F) and GC-2 (H) cells. Scale bar = 50 μm for RAW264.7, 100 μm for GC-2. (G, I) Quantitative analysis of reduced/oxidized BODIPY-C11 fluorescence intensity ratio in RAW264.7 (G) and GC-2 (I) cells. (J) Immunofluorescence images of GPX4 expression in GC-2 cells. Scale bar = 100 μm. (K) Quantification of GPX4 mean fluorescence intensity (MFI) in GC-2 cells. Data are presented as mean ± SD, n = 3. Statistical significance is indicated as ns, not significant; *P < 0.05; ***P < 0.001; ****P < 0.0001.

Given that mitochondria are the primary source of ROS and sustained overproduction disrupts mitochondrial membrane potential (ΔΨm), forming a vicious oxidative amplification cycle [40], we further decipher the cytoprotective mechanism of ZnTFZCDs against MPs toxicity at the mitochondrial level in both cell lines (schematic diagram in Fig. 4A). JC-1 staining was first employed to evaluate mitochondrial membrane potential (ΔΨm) in RAW264.7 and GC-2 cells, where red fluorescence indicates JC-1 aggregates for high mitochondrial membrane potential and green fluorescence represents JC-1 monomers for low mitochondrial membrane potential. Control cells displayed intact mitochondrial polarization with strong JC-1 red fluorescence, whereas MPs exposure markedly shifted fluorescence to green and reduced the aggregate/monomer ratio in both cell lines (Fig. 4B–E), confirming substantial mitochondrial depolarization. Treatment with TFZ or TFZCDs partially restored ΔΨm, whereas ZnTFZCDs produced the most pronounced recovery, as evidenced by the reappearance of red fluorescence and a markedly increased JC-1 ratio. The mitochondrial ΔΨm collapse caused by MPs is particularly deleterious to germ cells given their high energy demand and reliance on redox homeostasis [14], whereas the pronounced mitochondrial preservation by ZnTFZCDs, mechanistically underpins their cytoprotective action and may contribute to the interception of cuproptotic pathways, given the central role of mitochondrial dysfunction in copper-driven cell death [41].

Having established that ZnTFZCDs preserve mitochondrial membrane potential, we next examined whether this mitochondrial protection translates into reduced oxidative damage to membrane lipids. MPs exposure markedly increased green fluorescence while decreasing red fluorescence in both cell lines, with a significantly reduced reduced/oxidized ratio, confirming severe lipid peroxidation (Fig. 4F–I). Notably, ZnTFZCDs treatment effectively restored the red/oxidized ratio relative to TFZ and TFZCDs, suggesting that the cytoprotective effect involves mitigation of MP-induced lipid peroxidation. To determine whether this recovery reflects reduced ROS production and/or accelerated lipid peroxide clearance, we assessed the expression of GPX4, the principal enzyme that reduces phospholipid hydroperoxides within cellular membranes [42]. Consistently with our *in vivo* western blot results, MPs exposure markedly downregulated GPX4 fluorescence intensity, revealing GPX4 depletion as a critical driver for unchecked lipid peroxidation, whereas ZnTFZCDs treatment prominently upregulated GPX4 protein expression, replenishing cellular GPX4 reservoir to constrain lipid peroxidation progression (Fig. 4J and K). Collectively, ZnTFZCDs exert multi-layered cytoprotection against MPs toxicity by stabilizing mitochondrial membrane potential to curb ROS overproduction, reversing GPX4 depletion to suppress lipid peroxidation, and reconstructing cellular redox homeostasis. The superior activity of ZnTFZCDs arises from synergistic effects of carbon dot structural engineering and zinc doping through the TFZ-derived carbon scaffold with enlarged surface area and phenolic hydroxyl sites for radical trapping and GPX4 stabilization [43], together with zinc incorporation that optimizes electron transfer and likely confers mitochondrial targeting and ROS-quenching capacity [44,45], collectively preserving spermatogenic cell integrity.

### 2.5 ZnTFZCDs restore copper trafficking and metabolic protein homeostasis

Having verified MPs-induced disruption of testicular copper homeostasis *in vivo*, we applied TTM, Ferrostatin-1, 3-MA, and MCC950, pharmacological inhibitors targeting cuproptosis, ferroptosis, autophagy, and NLRP3-pyroptosis respectively, to evaluate their relative roles in GC-2 cells. TTM exerted the strongest protective effect, Ferrostatin-1 provided partial rescue, whereas 3-MA and MCC950 failed to restore cell viability following PS-MPs challenge (Fig. S7), indicating that cuproptosis is the predominant mediator of GC-2 cell injury, while ferroptosis contributes only modestly and autophagy and NLRP3-pyroptosis show negligible impact on cell viability under present experimental conditions.

**Fig. 5:**
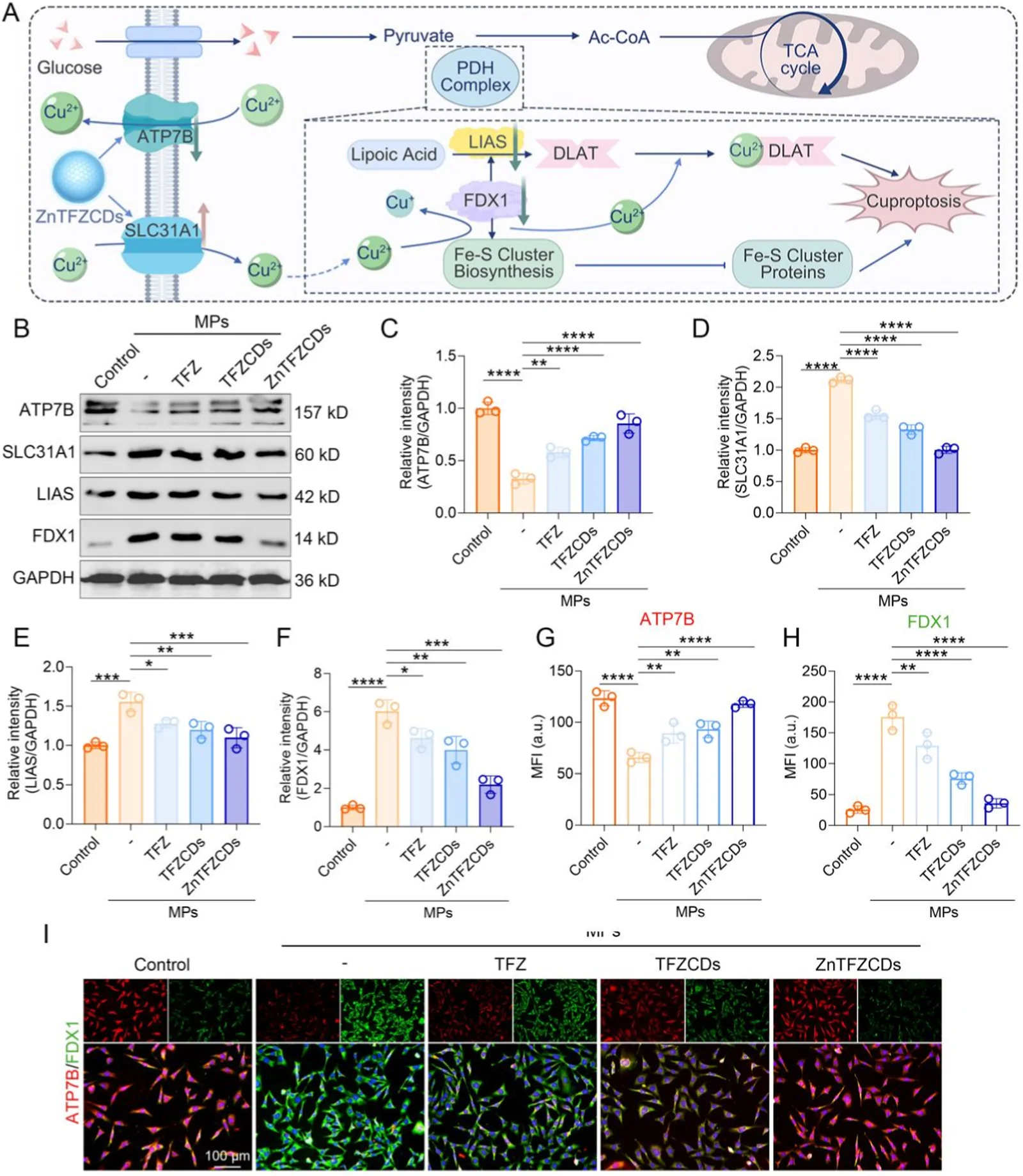
**ZnTFZCDs reverse MPs-induced dysregulation of copper transport and metabolism-related proteins in GC-2 cells.** (A) Schematic summary illustrating the molecular mechanism by which ZnTFZCDs remodel MPs-disrupted copper homeostasis and downstream copper-mediated metabolic perturbation. (B-F) Western blot analysis (B) and quantitative analysis of cuproptosis-related proteins ATP7B (C), SLC31A1 (D), LIAS (E), and FDX1 (F). (G-I) Representative immunofluorescence images (I) and quantitative analysis of ATP7B (red, G) and FDX1 (green, H). Scale bar = 100 μm. Data are presented as mean ± SD, n = 3. Statistical significance is indicated as *P < 0.05, **P < 0.01, ***P < 0.001, and ****P < 0.0001.

Following these pharmacological assessments, we next focused on investigating whether ZnTFZCDs could restore MPs-disrupted copper transport and cuproptosis-associated metabolic signaling in GC-2 cells. Consistently, MPs exposure significantly downregulated copper efflux transporter ATP7B while markedly elevating the level of influx transporter SLC31A1 (Fig. 5B–D), a pattern driving excessive intracellular copper accumulation [46]. Meanwhile, MPs stimulation induced prominent upregulation of LIAS and FDX1 (Fig. 5B–E, F), two key proteins governing lipoic acid metabolism and Fe-S cluster biosynthesis, which have been established as proximal executors of cuproptosis [16]. Treatment with ZnTFZCDs intervention significantly rescued ATP7B expression, suppressed abnormal SLC31A1 overexpression, and normalized the elevated LIAS and FDX1 levels, effectively correcting MPs-induced imbalance in copper transport and downstream metabolic protein perturbation. Dual-colour immunofluorescence co-staining was adopted to separately visualize the expression level and intracellular distribution of ATP7B and FDX1 in GC-2 cells (Fig. 5G–I). Quantitative MFI statistics revealed that MPs treatment suppressed ATP7B fluorescence while drastically elevating FDX1 abundance, with ZnTFZCDs notably recovering ATP7B and alleviating FDX1 overexpression compared with TFZ and TFZCDs, highly consistent with western blot data. Copper overload suppresses the respiratory chain and depletes ATP, leading to ROS burst, which in turn oxidizes thiol groups of lipoylated proteins and enhances copper-DLAT binding affinity, thereby creating a self-amplifying cycle of mitochondrial injury [47]. ZnTFZCDs counteract this cycle by alleviating MPs-induced mitochondrial oxidative damage, rebalancing copper influx/efflux to correct copper metabolic disorder, and reversing aberrant LIAS/FDX1 upregulation, which suppresses DLAT lipoylation and mitigates proteotoxic stress (Fig. 5A). Nevertheless, these *in vitro* observations require systematic *in vivo* investigation to ascertain the comprehensive protective profile of ZnTFZCDs under physiologically relevant conditions.

### 2.6 ZnTFZCDs ameliorate reproductive injury-related molecular dysfunction

**Fig. 6:**
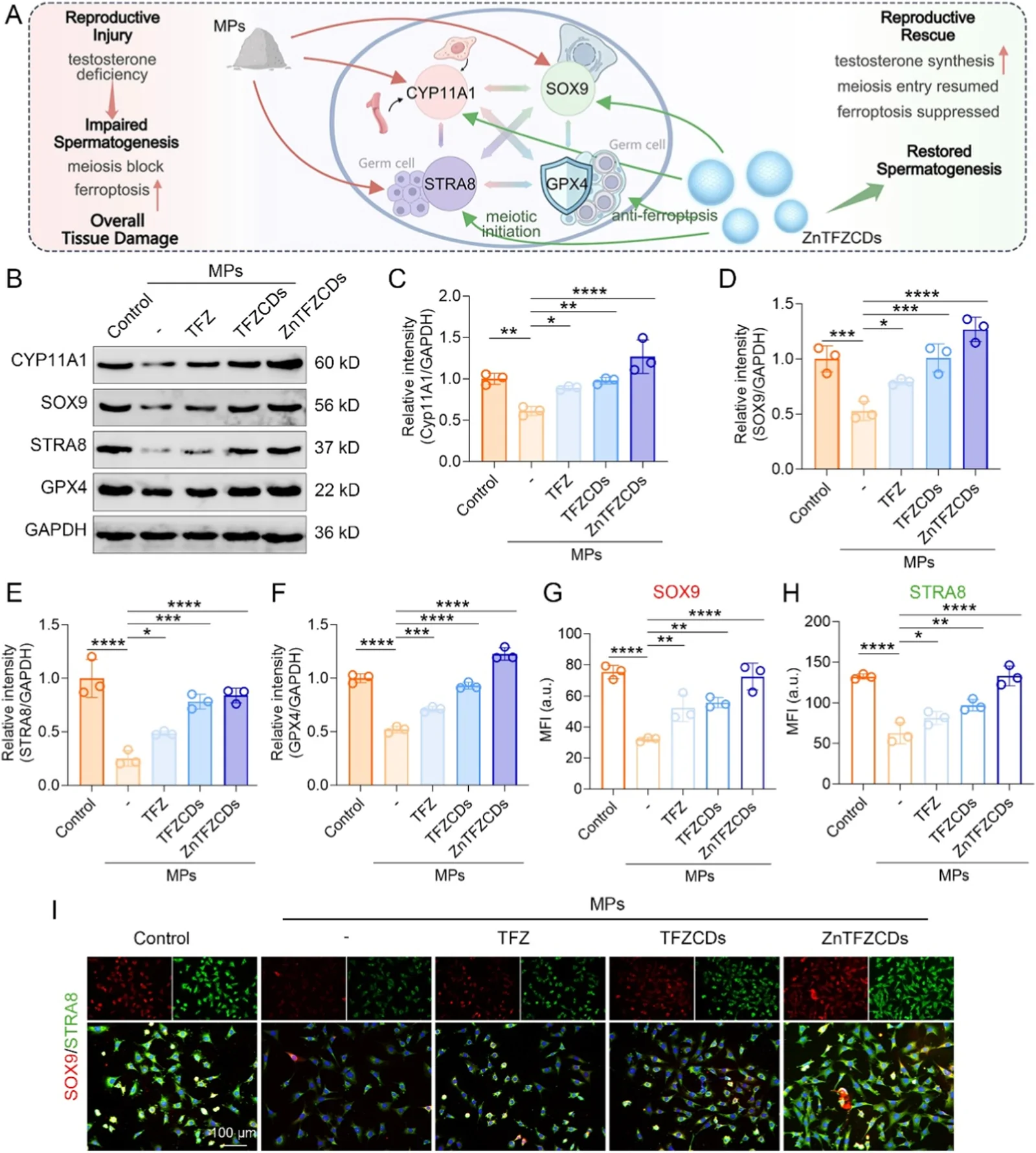
**ZnTFZCDs rescue MPs-induced suppression of steroidogenic proteins and meiotic initiation-related transcription factors in GC-2 cells.** (A) Schematic overview illustrating the protective cascade of ZnTFZCDs against MPs-mediated reproductive toxicity. (B-F) Western blot analysis (B) and quantitative analysis of CYP11A1 (C), SOX9 (D), STRA8 (E), and GPX4 (F). (G-I) Representative immunofluorescence images (I) and quantitative analysis of SOX9 (red, G) and STRA8 (green, H). Scale bar = 100 μm. Data are presented as mean ± SD, n = 3. Statistical significance is indicated as *P < 0.05, **P < 0.01, ***P < 0.001, and ****P < 0.0001.

To determine whether the redox-protective and anti-cuproptotic effects of ZnTFZCDs translate into preservation of spermatogenic molecular programs, a panel of reproductive function-associated proteins was examined, as summarized in the mechanistic scheme shown in Fig. 6A. MPs exposure significantly downregulated CYP11A1, SOX9, STRA8, and GPX4 in GC-2 cells, corroborating the *in vivo* observations and confirming that the suppressive effects on steroidogenesis-related, Sertoli-supportive, meiotic, and antioxidant machinery are recapitulated at the cellular level. Notably, while all three treatments alleviated such aberrant protein expression to different extents, ZnTFZCDs exerted the strongest restorative effect, inducing more substantial recovery of CYP11A1, SOX9, STRA8 and GPX4 levels relative to free TFZ and TFZCDs (Fig. 6B–F). Meanwhile, immunofluorescence staining further confirmed the western blot findings, showing that MPs exposure markedly reduced the MFI of both SOX9 and STRA8, while ZnTFZCDs restored their fluorescence to near-control levels (Fig. 6G–I). Mechanistically, the restoration of these four proteins by ZnTFZCDs is likely a secondary consequence of upstream stress relief, which normalizes oxidative modification of transcription factors or epigenetic dysregulation by reversing ROS overproduction and mitochondrial dysfunction, thereby restoring steroidogenic enzymes and meiotic regulators [48,49]. Beyond this indirect pathway, zinc ions from ZnTFZCDs may restore GPX4 through two convergent mechanisms, namely facilitating NRF2-dependent selenium uptake for selenoprotein synthesis and stabilizing GPX4 mRNA to enhance its expression [50,51]. In turn, the restored GPX4 reinforces the antioxidant barrier and preserves mitochondrial function, curbing further ROS production and forming a forward-amplifying loop that sustains both the recovery of steroidogenic and meiotic regulators and the structural integrity of germ cells [52]. Thus, ZnTFZCDs are not merely antioxidant nanomaterials but multifunctional regulators that establish a complete axis from upstream stress alleviation to downstream functional recovery of the spermatogenic program.

### 2.7 ZnTFZCDs exert multi-target reproductive protection *in vivo*

**Fig. 7:**
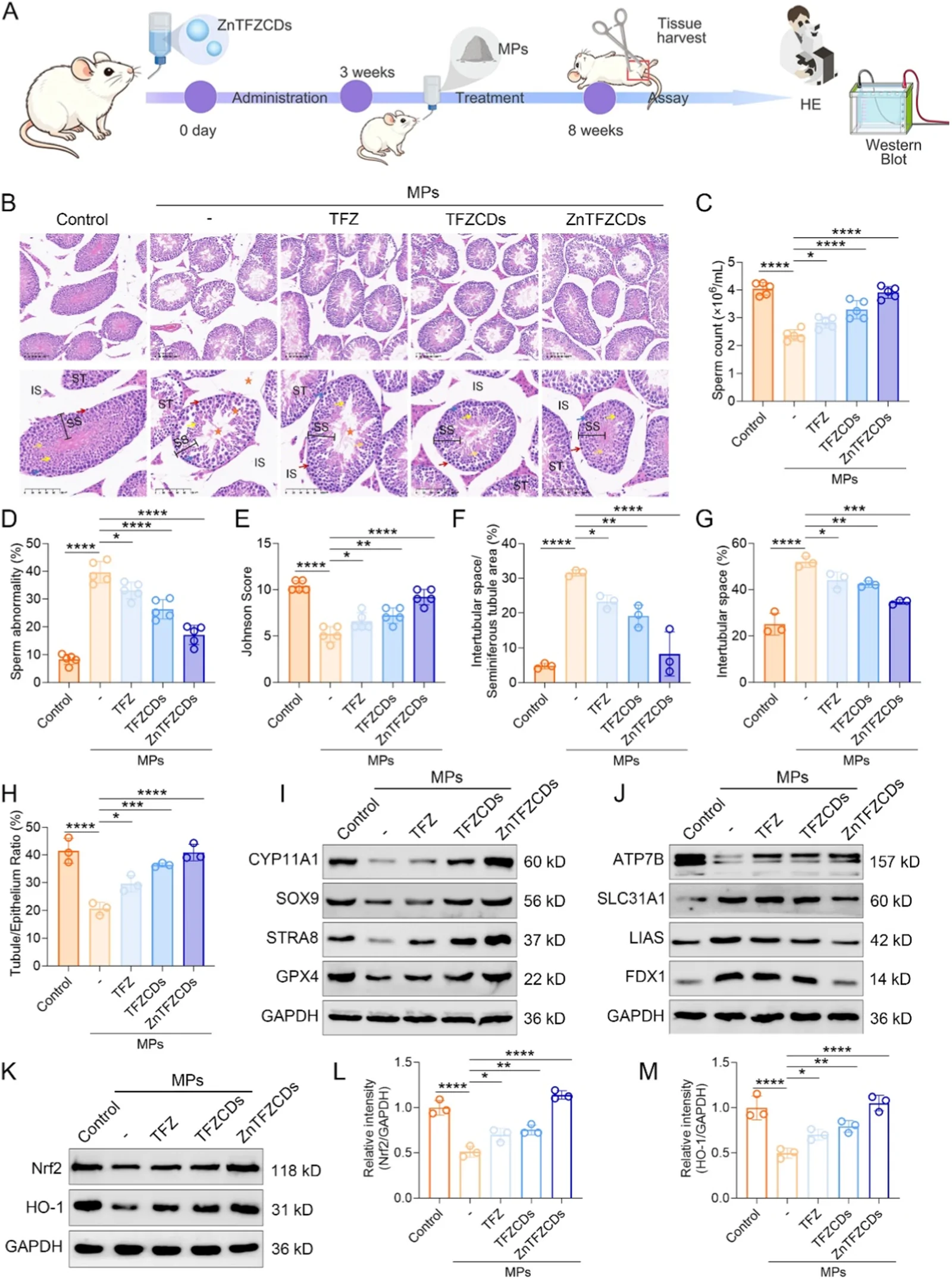
**ZnTFZCDs alleviate MPs-induced testicular injury, copper dyshomeostasis and suppressed Nrf2 antioxidant pathway *in vivo*.** (A) Schematic timeline of animal administration, MPs exposure, sample collection and subsequent detection. (B) Representative H&E staining images of testicular sections. Scale bar = 100 μm. (C-H) Quantitative statistics of epididymal sperm count (C), sperm abnormality rate (D), testicular Johnson score (E), Intertubular space/Seminiferous tubule area (F), intertubular space area (G), and tubule/epithelium ratio (H). (I, J) Western blot analysis of reproductive injury-related proteins CYP11A1, SOX9, STRA8, GPX4(I) and cuproptosis-related proteins ATP7B, SLC31A1, LIAS, FDX1 (J). (K-M) Western blot analysis (K) and quantitative analysis of oxidative stress-related proteins Nrf2 (L) and HO-1 (M) in testicular tissues. Data are presented as mean ± SD, biological replicates: n = 5 for C-E; n = 3 for F-H, L, M. Statistical significance is indicated as *P < 0.05, **P < 0.01, ***P < 0.001, and ****P < 0.0001.

To verify whether the protective effects observed *in vitro* could be translated into *in vivo* efficacy, an 8-week mouse exposure regimen was established to systematically verify the protective efficacy of ZnTFZCDs against MPs-triggered testicular damage and related molecular disorder (Fig. 7A). Histopathological observation via hematoxylin-eosin (HE) showed that the control group exhibited intact seminiferous tubules with a compact and well-organized seminiferous epithelium, and germ cells at different developmental stages were orderly arranged along the tubule wall (Fig. 7B). In contrast, MPs exposure caused prominent structural damage in the testis, including loosening and disorganization of the seminiferous epithelium, germ-cell exfoliation or loss, and marked enlargement of the seminiferous and interstitial spaces. Quantitative statistical analysis further confirmed that MPs treatment significantly reduced epididymal sperm count (Fig. 7C), raised sperm abnormality rate (Fig. 7D), lowered Johnson score reflecting spermatogenic integrity (Fig. 7E), elevated the ratio of interstitial area to seminiferous tubule area (Fig. 7F) and the percentage of interstitial space across total tissue area (Fig. 7G), and decreased the tubule-to-epithelium ratio (Fig. 7H). Consistent with *in vitro* trends, ZnTFZCDs treatment exerted more prominent protective effects than TFZ and TFZCDs, which preserved ordered seminiferous tubular architecture, mitigated germ cell shedding, elevated sperm quantity, decreased abnormal sperm ratio, improved Johnson score, and normalized interstitial and tubular structural parameters.

Western blot analysis of testicular tissues further confirmed the key molecular alterations observed *in vitro*. ZnTFZCDs treatment effectively maintained testicular expression of the spermatogenesis-related proteins CYP11A1, SOX9, STRA8, and GPX4, modulated copper homeostasis by reversing ATP7B downregulation and SLC31A1 upregulation while suppressing LIAS and FDX1 overexpression, and reactivated the Nrf2/HO-1 antioxidant axis (Fig. 7I–M, S8, 9). These regulatory effects were consistently stronger than those of TFZ and TFZCDs, confirming the superior *in vivo* efficacy of ZnTFZCDs in preserving spermatogenic molecular programs, copper homeostasis, and antioxidant defense. Reproductive toxicity caused by environmental pollutants is rarely mediated by a single pathway, but rather arises from interconnected and mutually reinforcing pathogenic events [53], suggesting that single-target strategies may have limited protective efficacy. By incorporating functional metal doping into natural product-derived carbon dots like ZnTFZCDs, this strategy enables these nanoplatforms to retain precursor bioactivity while serving as programmable multi-target intervention tools, offering a promising solution for complex environmental exposures and related health risks [54].

### 2.8 ZnTFZCDs exhibit favorable *in vitro* and *in vivo* biosafety

**Fig. 8:**
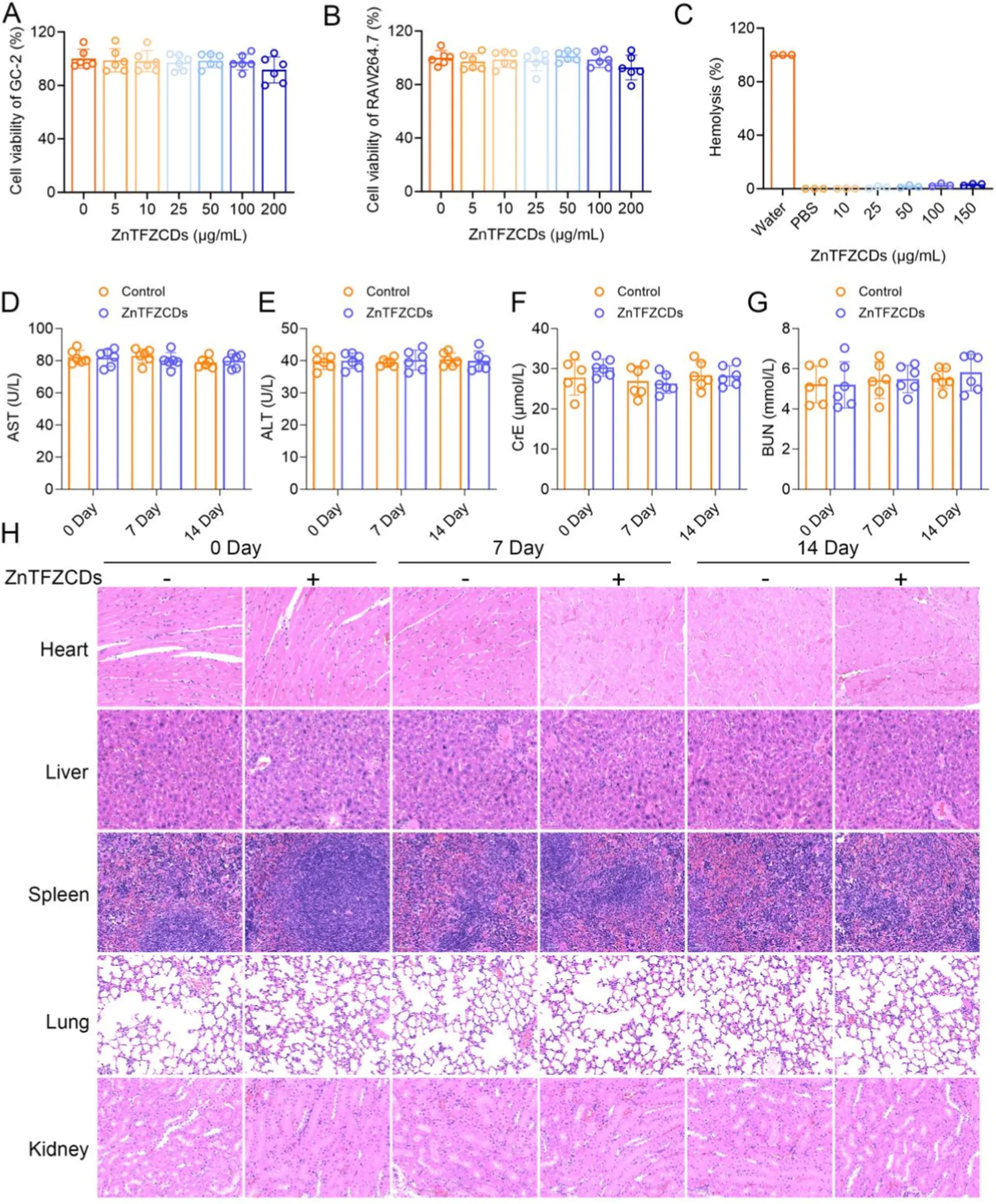
**Biosafety evaluation of ZnTFZCDs.** (A, B) Cell viability of GC-2 and RAW264.7 cells after incubation with different concentrations of ZnTFZCDs. (C) Hemolysis assay of ZnTFZCDs at different concentrations. Water was used as the positive control and PBS as the negative control. (D-G) Serum levels of AST, ALT, Cre and BUN in mice at day 0, day 7 and day 14 following ZnTFZCDs administration. (H) Representative H&E staining images of major organs, including heart, liver, spleen, lung, and kidney, collected from mice treated with or without ZnTFZCDs at 0, 7, and 14 days. Data are presented as mean ± SD, biological replicates: n = 6 for A, B, D-G; n = 3 for C.

For nanomaterials intended for biomedical intervention, especially for applications involving repeated administration and reproductive protection, a satisfactory biosafety profile is a fundamental prerequisite [55]. Therefore, the biocompatibility of ZnTFZCDs was systematically evaluated at both the cellular and animal levels. CCK-8 assays were implemented to evaluate the cytotoxicity profile of gradient concentrations of ZnTFZCDs toward GC-2 spermatogenic cells and RAW264.7 macrophages (Fig. 8A and B). No obvious decline in cell viability was observed across the tested concentration range from 0 to 200 μg/mL for both cell lines, demonstrating that ZnTFZCDs exhibit negligible intrinsic cellular toxicity within the experimental dosage window. Hemolysis assay was further conducted to assess blood compatibility (Fig. 8C). Distinct from the positive control group with severe hemolysis triggered by pure water, the hemolysis ratio remained extremely low at all tested ZnTFZCDs concentrations, confirming favorable hemocompatibility and which is an important safety feature for *in vivo* administration [56].

Subsequent *in vivo* systemic biosafety detection was carried out after continuous administration of ZnTFZCDs for 0, 7 and 14 days. Serum biochemical indices including AST, ALT, Cre and BUN were measured to reflect hepatic and renal function (Fig. 8D–G). No significant fluctuations in these liver and kidney injury markers were detected between the control group and ZnTFZCDs-treated group at all time points. Consistently, histological observation via HE staining of major visceral organs heart, liver, spleen, lung and kidney displayed intact tissue structure, regular cell arrangement and no visible inflammatory infiltration, necrosis or pathological lesion in ZnTFZCDs-treated mice relative to untreated controls. In summary, both *in vitro* and *in vivo* biosafety assessments confirm that ZnTFZCDs exhibit excellent biocompatibility, as evidenced by low cytotoxicity, negligible hemolysis, no disruption of serum biochemical parameters, and no histopathological damage to major organs. This favorable safety profile is particularly critical for the intended application in male reproductive protection, as it ensures that the observed therapeutic benefits against MPs-induced injury are not compromised by unintended systemic toxicity. Moreover, given the increasing prevalence of environmental MPs and their potential to accumulate in reproductive tissues, the ZnTFZCDs platform may offer a translatable strategy for long-term, repeated-administration scenarios. Future studies are warranted to evaluate its biodistribution, metabolic fate, and efficacy in more clinically relevant models, such as non-human primates or chronic low-dose exposure regimes.

## 3 Conclusion

In this study, we found that chronic MPs exposure induces redox imbalance, copper dyshomeostasis, testicular damage and spermatogenic dysfunction, prompting us to develop zinc-doped trifolirhizin-derived carbon dots (ZnTFZCDs) that target these pathways for reproductive injury treatment. Mechanistically, ZnTFZCDs effectively scavenge excessive ROS, maintain antioxidant enzyme activities, preserve ΔΨm and upregulate GPX4 expression, thereby breaking the mitochondrial-driven lipid peroxidation cascade and alleviating oxidative damage. Moreover, ZnTFZCDs modulate copper homeostasis by upregulating the copper exporter ATP7B, downregulating the influx transporter SLC31A1, and suppressing the cuproptosis executors FDX1 and LIAS. In addition to excellent biocompatibility, ZnTFZCDs improved reproductive-related protein profiles in GC-2 cells and further translated into improved sperm quality and testicular histopathology in the mouse model. Collectively, our findings establish ZnTFZCDs as a bio-inspired nanotherapeutic strategy that addresses MPs-induced male reproductive injury through coordinated modulation of redox and copper homeostasis, offering a new paradigm for combating environmental pollutant-related reproductive disorders. However, this study mainly demonstrates the preventive and protective effects of ZnTFZCDs *in vivo*. Future studies are warranted to evaluate the long-term biodistribution, metabolic fate, and therapeutic potential of ZnTFZCDs in clinically relevant injury models, as well as to explore their broader applicability against other environmental toxicant-induced reproductive impairments.

## 4 Material and methods

### 4.1 Reagents and antibodies

Trifolirhizin (TFZ, T828362) and zinc nitrate hexahydrate (Zn(NO_3_)_2_·6H_2_O, Z118841) were purchased from Macklin (Shanghai, China) and Aladdin (Shanghai, China), respectively. Microplastics (MPs, spherical shape, average diameter 5 μm) were supplied by Peiseler Chromatography Technology Development Centre (Tianjin, China). The CCK-8 cell viability detection kit (CA1210), DCFH-DA (D6470), mitochondrial membrane potential detection reagent JC-1 (M8650), assay kits used for detection of CAT (BC0205), SOD (BC5165), GPx (BC1195), MDA (BC0025), Cre (BC4915), BUN (BC1535) and liver function (AST + ALT) assay kit (BC-S001), were all supplied by Solarbio (Beijing, China). BODIPY lipid peroxidation fluorescent probe (SML3717) was purchased from Sigma-Aldrich (USA).

Primary antibodies against CYP11A1 (13363-1-AP), SOX9 (55152-1-AP), STRA8 (68071-1-lg), GPX4 (30388-1-AP), ATP7B (19786-1-AP), SLC31A1 (27499-1-AP), LIAS (11577-1-AP), FDX1 (12592-1-AP), Nrf2 (16396-1-AP), HO-1 (10701-1-AP),GAPDH (10494-1-AP) and Horseradish peroxidase conjugated goat anti-rabbit (RGAR001) and anti-mouse (RGAM001) were purchased from Proteintech (Wuhan, China). IFluor 594 labeled goat anti-rabbit (HA1122) and IFluor 488 labeled goat anti-mouse (HA1125) used for immunofluorescence staining were all obtained from HUABIO (Hangzhou, China).

### 4.2 Synthesis and physicochemical characterization of ZnTFZCDs

Zn-doped trifolirhizin-derived carbon dots (ZnTFZCDs) were synthesized via a one-pot hydrothermal reaction. The synthesis of Zn-doped carbon dots follows a bioinspired strategy [57,58]. Histidine, a natural metal-chelating amino acid in metalloproteins, was selected as precursor to simulate biological Zn-coordination microenvironment for carbon dot construction. Briefly, 20 mg of TFZ was dispersed in 1 mL of ultrapure water by ultrasonication for 5 min to obtain a homogeneous suspension. Meanwhile, 100 mg of citric acid and 20 mg of zinc nitrate hexahydrate were dissolved in 18 mL of ultrapure water under magnetic stirring. The TFZ suspension was then slowly added into the above solution, followed by the addition of 100 μL of ethylenediamine as a nitrogen source. The mixed precursor solution was adjusted to pH 7.5-8.5 with NaOH, transferred into a 50 mL Teflon-lined stainless-steel autoclave, and heated at 180 °C for 6 h. After natural cooling to room temperature, the resulting brownish-yellow product was filtered through a 0.22 μm microporous membrane to remove large particulates. The filtrate was then dialyzed against ultrapure water using a dialysis bag with a molecular weight cutoff of 3000 Da for 24-48 h to remove unreacted small molecules, free zinc ions, and other impurities. Finally, the purified solution was lyophilized to obtain ZnTFZCDs as a solid powder. For comparison, undoped TFZCDs were prepared following the same procedure without the addition of zinc acetate dihydrate.

The morphological and structural features of ZnTFZCDs were systematically characterized. TEM and HRTEM were applied to observe the particle morphology, dispersion state and lattice fringe structure of ZnTFZCDs. DLS was used to detect the hydrodynamic particle size distribution, while zeta potential measurement was performed to analyze the surface charge properties of TFZ, TFZCDs and ZnTFZCDs. UV-vis spectra were recorded to identify the characteristic absorption peaks of raw trifolirhizin and synthesized ZnTFZCDs. XRD patterns were collected to analyze the carbon skeleton crystallinity of ZnTFZCDs. FTIR spectroscopy was adopted to distinguish the surface functional groups of TFZ and ZnTFZCDs. XPS full survey and high-resolution C 1s, O 1s, N 1s, Zn 2p spectra were acquired to confirm elemental composition and surface chemical bonding states of ZnTFZCDs.

### 4.3 Establishment of *in vivo* MPs exposure model and sample collection

Male ICR mice (6-8 weeks old) were obtained from the Laboratory Animal Center of Xuzhou Medical University (XZMU, Xuzhou, China) and bred in a SPF facility under controlled temperature (22 ± 2 °C) and humidity (50 ± 10%), with a 12-h light/12-h dark cycle and free access to standard chow and sterilized water. All mice randomly assigned into five groups including control group, MPs group, MPs plus free TFZ group, MPs plus TFZCDs group and MPs plus ZnTFZCDs group. An eight-week experimental protocol was conducted for animal modeling and therapeutic evaluation. Briefly, mice were orally administered with ZnTFZCDs, TFZCDs, or free TFZ for the first three weeks, followed by five weeks of co-administration with MPs suspension to establish the testicular reproductive injury mode. At the end of the experimental cycle, mice were anesthetized and sacrificed, and the epididymides and testicular tissues were rapidly dissected. Sperm count and sperm abnormality rate were determined from epididymal homogenates. Portions of testicular tissues were fixed for hematoxylin-eosin staining and pathological quantitative analysis, while the remaining samples were snap-frozen in liquid nitrogen and stored at −80 °C for total protein extraction and subsequent immunoblot analysis. All animal procedures were approved by the Animal Ethics Committee of Jiangsu Normal University and performed in accordance with the relevant guidelines and regulations.

### 4.4 Testicular histopathology and quantitative morphometric analysis

Fixed testicular tissues were subjected to dehydration, paraffin embedding and slicing before routine hematoxylin-eosin staining operation. Optical microscope was used to observe histological morphology of seminiferous tubules, arrangement status of spermatogenic cells and structural changes of testicular interstitial area. Multiple quantitative indicators were statistically analyzed including epididymal total sperm count, sperm abnormality rate, Johnsen scoring reflecting spermatogenic integrity, area ratio of interstitial tissue relative to seminiferous tubule, percentage of interstitial area accounting for total visual field area and tubule epithelium ratio. Statistical measurement was conducted from multiple randomly selected visual fields to ensure objectivity of quantitative results.

### 4.5 Cell culture and viability assessment

Mouse spermatogenic GC-2 cells and mouse mononuclear macrophage RAW264.7 cells were cultured in DMEM-High Glucose medium and RPMI 1640, respectively, with 10% fetal bovine serum, and 1% penicillin-streptomycin. Cells were maintained in a humidified incubator with 5% carbon dioxide concentration at 37 °C. Two independent CCK-8 experiments were conducted for different research purposes. For cytoprotective comparison under microplastic exposure, GC-2 cells and RAW264.7 cells were seeded into 96-well culture plates at consistent density and cultured overnight to complete cell adherence treatment, and cellular damage was induced by prior MPs stimulation before treatment with equal concentrations of TFZ, TFZCDs and ZnTFZCDs respectively for another 24 h. For intrinsic biosafety detection of the material itself, cells were seeded and attached in the same way, and ZnTFZCDs with concentration gradients set as 0, 5, 10, 25, 50, 100 and 200 μg/mL were added into each well for 24 h of incubation. Ten microliters of CCK-8 working solution was supplemented into each well followed by 1.5 h of incubation under dark environment. The absorbance value at 450 nm was detected with a microplate reader, and relative cell viability was calculated with the untreated cell group defined as 100% viability.

### 4.6 Intracellular ROS and oxidative stress related index detection

DCFH-DA fluorescent probe was utilized to quantify intracellular ROS accumulation in treated GC-2 and RAW264.7 cells. After scheduled intervention, cells were washed repeatedly with pre-cooled phosphate-buffered saline. DCFH-DA working solution was then added for light-shielded incubation at 37 °C for 40 min. Fluorescence images were captured using a fluorescence microscope, and the corresponding fluorescence intensity was statistically quantified. Cell lysates were collected from different treatment groups, including those treated with TFZ, TFZCDs, and ZnTFZCDs, to determine the activities of CAT, SOD, and GPx, as well as MDA content. All detection operations were performed strictly in accordance with the instructions of the corresponding commercial test kits. Absorbance data at specific wavelengths were collected using a microplate reader, and the final results were normalized to the total protein concentration of each cell sample.

### 4.7 Mitochondrial membrane potential and lipid peroxidation fluorescent staining

JC-1 fluorescent probe was employed to evaluate mitochondrial membrane potential changes in GC-2 and RAW264.7 cells following MPs and material interventions. After the scheduled treatments, cells were gently washed with pre-cooled PBS and incubated with the prepared JC-1 working solution at 37 °C for 20 min in the dark. Fluorescence images were captured using a fluorescence microscope to collect both red aggregate and green monomer fluorescence signals. The ratio of red to green fluorescence intensity was then calculated to determine the degree of mitochondrial depolarization. In parallel, the BODIPY-C11 probe was utilized to label intracellular lipid peroxidation products.Following the corresponding treatments, cells were fixed with 4% paraformaldehyde at room temperature for 15 min, then incubated with BODIPY-C11 working solution (5 μM) at 37 °C for 30 min away from light, and finally counterstained with DAPI for 5 min at room temperature in the dark. Fluorescence intensity of the reduced and oxidized forms of the BODIPY-C11 probe was analyzed to assess the severity of intracellular lipid peroxidation injury.

### 4.8 Immunofluorescence staining

Three immunofluorescence staining schemes were performed on GC-2 spermatogenic cells, including single staining for GPX4 and dual co-staining for ATP7B/FDX1 and SOX9/STRA8. Cells were seeded on sterile slides, subjected to designated treatments, fixed with 4% paraformaldehyde, permeabilized with 0.1% Triton X-100 for 20 min, and blocked with 5% BSA. For single staining, slides were incubated with anti-GPX4 primary antibody overnight at 4 °C, followed by the corresponding fluorescent secondary antibody in the dark. For dual co-staining, slides were incubated overnight with a mixture of two species-matched primary antibodies (anti-ATP7B plus anti-FDX1, or anti-SOX9 plus anti-STRA8), and then with two corresponding species-specific fluorescent secondary antibodies under light-shielded conditions. DAPI was used for nuclear counterstaining in all groups before mounting. All fluorescence images were captured using a fluorescence microscope, and mean fluorescence intensity of target proteins was quantified with ImageJ software. Quantitative colocalization analysis was not performed.

### 4.9 Western blot analysis

Total protein was extracted from cultured GC-2 cells and harvested mouse testicular tissue specimens using RIPA lysis buffer supplemented with 100× protease inhibitor cocktail. The total protein concentration of each sample was determined using a BCA protein quantification kit. Equal amounts of protein were separated by sodium dodecyl sulfate-polyacrylamide gel electrophoresis and then transferred onto 0.22 μm PVDF membranes. The membranes were blocked with 5% skim milk at room temperature for 2 h to prevent non-specific binding. They were then incubated overnight at 4 °C with primary antibodies against CYP11A1, SOX9, STRA8, GPX4, ATP7B, SLC31A1, LIAS, FDX1, Nrf2, HO-1, and GAPDH. After thorough washing with TBST, the membranes were incubated with the corresponding horseradish peroxidase-conjugated secondary antibodies for 1 h at room temperature. Protein bands were visualized using enhanced chemiluminescence substrate. Gray-scale quantification of all target bands was performed using ImageJ software.

### 4.10 Comprehensive biosafety evaluation of ZnTFZCDs

Hemocompatibility test was conducted for *in vitro* biosafety evaluation. Fresh whole blood was collected from healthy experimental mice, and erythrocyte pellets were separated by centrifugation and washed repeatedly with pre-cooled phosphate-buffered saline. The erythrocyte suspension was then incubated with gradient concentrations of ZnTFZCDs solution at 37 °C for 1 h. Ultrapure water-treated erythrocyte suspension served as the positive hemolysis control, while PBS-treated suspension served as the negative control. After centrifugation, the absorbance of the supernatant at 545 nm was measured, and the hemolysis ratio of each group was calculated accordingly.

For systemic *in vivo* biosafety assessment, ZnTFZCDs solution was continuously administered to mice for 0, 7, and 14 days. At each preset time point, peripheral blood samples were collected and serum was separated. Commercial assay kits were used to measure serum levels of ALT, AST, Cre, and BUN to evaluate liver and kidney function. After blood collection, mice were sacrificed, and major visceral organs including the heart, liver, spleen, lung, and kidney were dissected and fixed in 4% paraformaldehyde. The tissues were embedded in paraffin, sectioned, and stained with hematoxylin and eosin. Histopathological changes in each organ were observed under an optical microscope to assess the systemic biocompatibility of ZnTFZCDs.

### 4.11 Statistical analysis

All experimental quantitative data were expressed as mean ± SD. GraphPad Prism software was used for all statistical analyses. Unpaired Student's t-test was applied to compare differences between two independent groups. One-way analysis of variance (ANOVA) followed by Tukey's post-hoc multiple comparison test was used for statistical comparisons among three or more groups. Statistical significance was denoted as follows: ns, not significant; *P < 0.05; **P < 0.01; ***P < 0.001; ****P < 0.0001.

## CRediT authorship contribution statement

**Tushuai Li:** Writing – original draft, Validation, Investigation, Data curation. **Kehui Li:** Writing – original draft, Validation, Investigation, Data curation. **Shiqing Yang:** Writing – original draft, Validation, Investigation, Data curation. **Hongying Pan:** Validation, Investigation, Data curation. **Zhihui Cai:** Validation, Investigation, Data curation. **Xiyao Hua:** Validation, Investigation, Data curation. **Guangfu Liao:** Writing – review & editing, Supervision, Funding acquisition, Conceptualization. **Bing Luo:** Writing – review & editing, Supervision, Funding acquisition, Conceptualization. **Jie Zhang:** Writing – original draft, Supervision, Funding acquisition, Conceptualization.

## Declaration of competing interest

The authors declare that they have no known competing financial interests or personal relationships that could have appeared to influence the work reported in this paper.

## Data Availability

Data will be made available on request.
