# Supplementary material for: Bioinspired zinc-doped carbon dots protect against polystyrene microplastic-induced spermatogenic dysfunction through Nrf2 activation and cuproptosis suppression
Source: Mater Today Bio. 2026 Sep 15;41:103675. doi: 10.1016/j.mtbio.2026.103675 (PMC13598172; doi:10.1016/j.mtbio.2026.103675)
Supplement: Supplementary file 1 [file mmc1.docx]

**Supporting information**

**Bioinspired zinc-doped carbon dots protect against polystyrene microplastic-induced spermatogenic dysfunction through Nrf2 activation and cuproptosis suppression**

Tushuai Li^1,#^, Kehui Li^1,#^, Shiqing Yang^2,#^, Hongying Pan^1^, Zhihui Cai^1^, Xiyao Hua^1^, Guangfu Liao^3,^*, Bing Luo^1,^*, Jie Zhang^1,^*

^1^ School of Biology and Food Engineering, Suzhou University of Technology, Suzhou, 215500, Jiangsu, China;

^2^ School of Life Science, Jiangsu Normal University, Xuzhou, 221116, Jiangsu, China;

^3^ College of Materials Engineering, Fujian Agriculture and Forestry University, Fuzhou 350002, China.

^#^These authors contributed equally.

***Correspondence:**

**Guangfu Liao**

Email: liaogf@fafu.edu.cn

**Bing Luo**

Email: binglou@szut.edu.cn

**Jie Zhang**

Email: [zhangj23@cslg.edu.cn](mailto:zhangj23@cslg.edu.cn)

**Figure S1. Quantitative analysis of spermatogenic and lipid peroxidation-related protein expression after gradient MPs exposure.** (A-D) Relative protein abundance of CYP11A1 (A), SOX9 (B), STRA8 (C), and GPX4 (D) in testicular tissues from mice subjected to oral administration of 0, 0.1, 1, and 10 mg/kg/d MPs. Data are presented as mean ± SD, n = 3. Statistical significance: ns, not significant; **P < 0.05, **P < 0.01, ***P < 0.001, ****P < 0.0001*.

**Figure S2. Quantitative analysis of copper homeostasis and cuproptosis-related protein expression after gradient MPs exposure.** (A-D) Relative protein abundance of ATP7B (A), SLC31A1 (B), LIAS (C), and FDX1 (D) in testicular tissues. Data are presented as mean ± SD, n = 3. Statistical significance: **P < 0.05, **P < 0.01, ***P < 0.001, ****P < 0.0001.*

**Figure S3. Quantitative analysis of Nrf2 antioxidant pathway-related protein expression after gradient MPs exposure.** (A-B) Relative protein abundance of Nrf2 (A) and HO-1 (B) in testes. Data are presented as mean ± SD, n = 3. Statistical significance: **P < 0.05, **P < 0.01, ***P < 0.001, ****P < 0.0001*.

**Figure S4. Cell viability of GC-2 cells after PS‑MPs insult with different treatments.** Cell viability was determined by CCK‑8 assay. Data are presented as mean ± SD, n = 3. ns, no significance; **P < 0.01, ****P < 0.0001.

**Figure S5. Concentration-dependent cytoprotective effects of ZnTFZCDs against MPs‑induced cytotoxicity.** (A, B) Cell viabilities of RAW264.7 (A) and GC-2 (B) were measured by CCK‑8 assay. Data are presented as mean ± SD, n = 3. **P < 0.05, ***P < 0.001, ****P < 0.0001*.

**Figure S6. Cytoprotective effects of ZnTFZCDs against PS-MP- and PE-MP-induced cytotoxicity in GC-2 cells.** GC-2 spermatogenic cells were exposed to PS-MPs or PE-MPs with or without ZnTFZCDs treatment. Cell viability was determined by CCK-8 assay. Data are presented as mean ± SD, n = 3. ***P < 0.01, ****P < 0.0001*.

**Figure S7. Pharmacological inhibitor rescue assay to evaluate different regulated cell‑death pathways in PS‑MPs‑treated GC-2 cells.** Cell viability was quantified by CCK‑8 assay. Data are presented as mean ± SD, n = 3. Statistical significance: ns, not significant; ***P < 0.01, ****P < 0.0001*.

**Figure S8. Quantitative analysis of spermatogenic function-related protein expression upon TFZ, TFZCDs, and ZnTFZCDs intervention in MPs-injured testes.** (A-D) Relative protein abundance of Cyp11A1 (A), STRA8 (B), SOX9 (C), and GPX4 (D). Data are presented as mean ± SD, n = 3. Statistical significance: ns, not significant; **P < 0.05, **P < 0.01, ***P < 0.001, ****P < 0.0001*.

**Figure S9. Quantitative analysis of copper homeostasis-related protein expression upon TFZ, TFZCDs, and ZnTFZCDs intervention in MPs-injured testes.** (A-D) Relative protein abundance of ATP7B (A), SLC31A1 (B), LIAS (C), and FDX1 (D). Data are presented as mean ± SD, n = 3. Statistical significance: ns, not significant; **P < 0.05, **P < 0.01, ***P < 0.001, ****P < 0.0001*.
